# Blended learning with Moodle in medical statistics: an assessment of knowledge, attitudes and practices relating to e-learning

**DOI:** 10.1186/s12909-017-1009-x

**Published:** 2017-09-19

**Authors:** Li Luo, Xiaohua Cheng, Shiyuan Wang, Junxue Zhang, Wenbo Zhu, Jiaying Yang, Pei Liu

**Affiliations:** 1 School of public health, Southeast University, Nanjing, 87 Dingjiaqiao China; 2 School of Computer Science and Engineering, Southeast University, Nanjing, China

**Keywords:** Blended learning, Curriculum development, E-learning, Medical statistics, Teaching reform

## Abstract

**Background:**

Blended learning that combines a modular object-oriented dynamic learning environment (Moodle) with face-to-face teaching was applied to a medical statistics course to improve learning outcomes and evaluate the impact factors of students’ knowledge, attitudes and practices (KAP) relating to e-learning.

**Methods:**

The same real-name questionnaire was administered before and after the intervention. The summed scores of every part (knowledge, attitude and practice) were calculated using the entropy method. A mixed linear model was fitted using the SAS PROC MIXED procedure to analyse the impact factors of KAP.

**Results:**

Educational reform, self-perceived character, registered permanent residence and hours spent online per day were significant impact factors of e-learning knowledge. Introversion and middle type respondents’ average scores were higher than those of extroversion type respondents. Regarding e-learning attitudes, educational reform, community number, Internet age and hours spent online per day had a significant impact. Specifically, participants whose Internet age was no greater than 6 years scored 7.00 points lower than those whose Internet age was greater than 10 years. Regarding e-learning behaviour, educational reform and parents’ literacy had a significant impact, as the average score increased 10.05 points (*P* < 0.0001).

**Conclusions:**

This educational reform that combined Moodle with a traditional class achieved good results in terms of students’ e-learning KAP. Additionally, this type of blended course can be implemented in many other curriculums.

## Background

Medical statistics is an applied science that combines statistical principles with methods of data collection, collation, analysis, and inference with their applications in medical research [1]. For most medical students, it is a compulsory curriculum. Although medical statistics is considered to be a useful tool in scientific research, students still cannot apply their knowledge in many situations such as study design and data analysis [2]. Therefore, it is necessary for tutors to take measures to improve medical students’ application ability. In addition, a sizable proportion of medical students in universities work as interns in a hospital. It is necessary to make learning more convenient and flexible to accommodate their schedules.

Blended learning offers the advantage of increased flexibility to students via online course learning while simultaneously maintaining face-to-face contact with teachers and other students [3]. It is defined as an innovative form of traditional face-to-face instruction in combination with technology-mediated instruction [4]. Optimal learning is achieved through a careful combination of traditional teaching and online learning [5]. Blended learning is being adopted by an increasing number of institutions of higher education [6]. In addition, it is predicted to be the “new traditional model” or the “new normal” in higher education [7].

Stacey and Gerbic [8] suggested that students’ maturity might contribute to a positive perspective of blended learning as well as a high degree of self-discipline and initiative for learning [9]. Accordingly, graduate students might be more appropriate for the hybrid modality, given their educational needs and expectations, than undergraduate students [10].

The concept of Web2.0 was created by Tim O’Reilly Media while communicating with working partners in 2004 [11, 12]. Web2.0 is an emerging Internet model that emphasizes students’ attendance to the construction, sharing, application and feedback of online learning materials but passive acceptance of knowledge from the Internet [13, 14]. Additionally, it has a profound impact on contemporary education and teaching. Web2.0 is not a specific tool. Instead, it comprises a type of tools, including the Modular Object Oriented Dynamic Learning Environment (Moodle) in this study. Moodle has been chosen by many institutions as the online learning platform due to its ease of use [15]. Moodle is an open-source course management system that was developed by Martin Dougiamas based on the “constructivist theory”. Its most attractive aspect is that it enables the creation of flexibility and engagement in learning courses [16]. Moodle allows teachers to organize e-learning activities and create new content, assignments, exams, etc. and students to access digital materials, participate in discussions, collaborate via forums, chats, wikis and blogs, view their progress, etc. [15, 16]. It has been adapted into primary and middle school as well as college courses [17–19]. However, no study has applied this kind of blended learning to education in medical statistics.

In this study, the Moodle platform and a traditional class were applied to postgraduate medical statistics education to improve learning outcomes. Our previous research indicated that the academic record is positively correlated with the numbers of students’ login [20]. The primary aim of this study was to determine whether specific student characteristics were related to e-learning knowledge, attitudes and practices (KAP). The secondary aim was to compare KAP before and after the intervention.

## Methods

### Participants

In this study, 119 first-year graduate students who were majoring in medicine at Southeast University (P.R. China) during the autumn 2014 semester participated. All of them were in the same blended class. The students experienced traditional face-to-face lectures as well as online interactions based on the Moodle platform during the course. One teacher and 6 teaching assistants received training before the intervention.

Participants’ e-learning KAP was assessed using the same real-name questionnaire at two time points: before the start of the intervention and at the end of the last day of the intervention.

### Teaching contents and methods

The Moodle-based pedagogical intervention was conducted as follows.

#### Teacher training

Although the Moodle platform does not require advanced computer skills, given that it involves a new form of teaching, Moodle is relatively novel among teachers who are accustomed to on-site teaching. Before class, we taught the teachers the basic operations of the Moodle platform, including registering, perfecting and revising the user profile, uploading learning materials, creating a new discussion post and assigning homework.

#### Register

To ensure quality and standardized management, we used the student number and name to set up accounts on the Moodle platform so that every student had an identifiable and unique account.

#### Student training and baseline survey

Prior to the students’ beginning the blended course, basic operations such as registering, perfecting and revising the user profile, welcoming, submitting homework, completing the test, downloading learning materials, and creating a new discussion post were introduced. The questionnaire was administered on Moodle in the form of a text, and all the students were required to complete it within one week.

#### Interaction between students and teachers

During the course, the tutor uploaded the courseware, assigned tasks, corrected homework, and generated questions regularly in the discussion area. The students downloaded the courseware, finished and submitted their homework, participated in discussions, and took the initiative to create a new discussion topic. The teaching contents included the statistical description of qualitative data, statistical description of quantitative data, t-test, analysis of variance (ANOVA), chi-square test, rank sum test, linear regression and correlation.

#### Second survey

At the end of the last class, the teacher asked the students to complete the same questionnaire again. Given that the Moodle platform was created on the college server, the students could log in to Moodle via the campus network, which offered convenience to promote the use of Moodle.

As all the participants provided their real name, the researchers could contact them via e-mail if anything was missing, which ensured the effective rate of the questionnaire.

### Statistical analysis

The information was derived from Moodle after the completion of the two surveys. An aim of this study was to evaluate the change in students’ e-learning KAP. We analysed binary data, ranked data and quantitative data (including the functions of Internet TV and the awareness of ten applications) with the chi-square test, rank sum test and t-test, respectively. The summed scores of every part (knowledge, attitude and practice) were calculated using the entropy method [21–23].

We fitted the mixed linear model [24, 25] using the SAS PROC MIXED procedure to analyse the impact factors of KAP. The variables of interest included educational reform, gender, the number of communities that the student joined during the undergraduate experience (hereinafter, community number), self-perceived character, registered permanent residence, parents’ literacy, Internet age, and hours spent online per day. The significance level was set at 0.10. Variables without statistical significance were excluded, and then the model was re-fitted. Ultimately, we obtained the optimal model.

## Results

### Response rate

The students were interviewed using a 25-item questionnaire. Moodle recorded 119 students’ login information. In the baseline survey, 115 questionnaires were collected. Additionally, the valid response rate was 96.6%. In the second survey, 94 questionnaires were collected. Furthermore, the valid response rate was 79.0%. The number of students complete the two surveys was 93. That is, 116 students completed at least one survey, and one of them finished the second survey, rather than the baseline survey.

### Demographic characteristics of the students

The majority of the participants were studying clinical medicine, biological engineering, medical laboratory, medical imaging, nursing, etc. The male-to-female ratio was 0.45:1. Other characteristics are listed in Table 1.

**Table 1:** Demographic characteristics of the students (*n*, %)

Variable	Classification	Number of People (*N* = 116)
Gender	Male	36 (31.03)
	Female	80 (68.97)
Community number	0	27 (23.28)
	1–2	77 (66.38)
	≥3	12 (10.34)
Self-perceived character	Introversion type	16 (13.79)
	Middle type	79 (68.10)
	Extroversion type	21 (18.10)
Registered permanent residence	Urban	60 (51.72)
	Rural	56 (48.28)
Parents’ literacy	College or above	29 (25.00)
	Middle or high school	70 (60.34)
	Primary or below	17 (14.66)
Internet age	6 years or below	55 (47.41)
	7 to 9 years	33 (28.45)
	10 years or above	28 (24.14)
Hours spent online per day	Less than 1 h	8 (6.90)
	1 to 3 h	78 (67.24)
	3 to 5 h	22 (18.97)
	More than 5 h	8 (6.90)

### Knowledge of e-learning

This section consisted of ten questions, including those relating to awareness of Web2.0, functions of Internet TV (a multiple-choice item), awareness of ten applications (a multiple-choice item), registering, uploading pictures, welcoming, downloading learning materials, submitting homework, participating in discussions, and taking an exam (See Table 2).

**Table 2:** Knowledge of e-learning (*n*, %)

Item	Classification	Before (*N* = 115)	After (*N* = 94)	*x* ^2^	*P*
Awareness of Web2.0	Never heard of it	97 (84.35)	60 (63.83)	11.39	0.0007*
	Heard of it but not familiar with it	17 (14.78)	33 (35.11)		
	Familiar with it	1 (0.87)	1 (1.06)		
Functions of Internet TV	1–2	20 (17.39)	11 (11.70)	-3.00^a^	0.0031
	3–4	40 (34.78)	17 (18.09)		
	5	55 (47.83)	66 (70.21)		
Awareness of ten applications	1–5	14 (12.17)	13 (12.77)	−0.46 ^a^	0.6429
	6–8	95 (82.61)	75 (79.79)		
	9–10	6 (5.22)	6 (6.38)		
Registering	No	10 (8.70)	2 (2.13)	4.11	0.0423
	Yes	105 (91.30)	92 (97.87)		
Uploading pictures	No	11 (9.57)	6 (6.38)	0.70	0.4025
	Yes	104 (90.43)	88 (93.62)		
Welcoming	No	3 (2.61)	0 (0.00)	0.99	0.3208
	Yes	112 (97.39)	94 (100.00)		
Downloading learning materials	No	8 (6.96)	2 (2.13)	1.69	0.1931
	Yes	107 (93.04)	92 (97.87)		
Submitting homework	No	7 (6.09)	0 (0.00)	4.19	0.0407
	Yes	108 (93.91)	94 (100.00)		
Participating in discussions	No	9 (7.83)	7 (7.45)	0.01	0.9183
	Yes	106 (92.17)	87 (92.55)		
Taking an exam	No	6 (5.22)	1 (1.06)	1.62	0.2027
	Yes	109 (94.78)	93 (98.94)		

^a^Shows the result of the two-sample t-test

*We applied multiple comparisons on the basis of an adjusted α = 0·0167: *p* = 0·0006 for “Never heard of it” versus “Heard of it but not familiar with it”, *p* = 0.7343 for “Never heard of it” versus “Familiar with it”, and *p* = 0·6442 for “Heard of it but not familiar with it” versus “Familiar with it”

As shown in Table 2, students’ awareness of Web2.0 was very low. Most of them had not heard of it. However, after the intervention, their familiarity was significantly higher (*P* < 0.01). The five functions of network TV that were listed in the questionnaire included creating a personal account, creating a homepage, commenting, uploading a video, and playing a video back. The accuracy before the intervention was 47.83%. It rose to 70.21% (*P* < 0.01) after the intervention. Most students had heard of 6 to 8 of the applications. Only a few people had heard of fewer than 5. More than 90% of the students had mastered basic operations of Moodle at the beginning of the intervention, although significant improvement was observed in registering or submitting homework.

### Students’ attitudes towards e-learning

Students had very positive attitudes towards e-learning. Most of them acknowledged that Moodle helped them to learn medical statistics to a large extent, and the proportion increased significantly after the course (*P* < 0.05). The proportion of participants who reported a strong willingness to use Moodle as a learning tool rose from 69.57% to 81.91% (*P* < 0.05). After the blended course, students’ interest in e-learning also increased greatly (see Table 3).

**Table 3:** Students’ attitudes towards e-learning (*n*, %)

Item	Classification	Before (*N* = 115)	After (*N* = 94)	*x* ^2^	*P*
How much Moodle helped you	None	2 (1.74)	0 (0.00)	4.93	0.0265†
	A little	45 (39.13)	25 (26.60)		
	A lot	68 (59.13)	69 (73.40)		
Willingness of using Moodle	Not pleased	1 (0.87)	0 (0.00)	4.30	0.0381‡
	Just so so	34 (29.57)	17 (18.09)		
	Very glad	80 (69.57)	77 (81.91)		
Interest in e-learning	Low	32 (27.83)	15 (15.96)	3.59	0.0582
	Just so so	76 (66.09)	72 (76.60)		
	Strong	7 (6.09)	7 (7.45)		

†We applied multiple comparisons on the basis of an adjusted α = 0·0167: *p* = 0·2989 for “None” versus “A little”, *p* = 0.1588 for “None” versus “A lot”, and *p* = 0·0457 for “A little” versus “A lot”;

‡We applied multiple comparisons on the basis of an adjusted α = 0·0167: *p* = 0·4858 for “Not pleased” versus “Just so so”, *p* = 0.3296 for “Not pleased” versus “Very glad”, and p = 0·0507 for “Just so so” versus “Very glad”;

### Students’ e-learning behaviour

Only 18.26% of the participants’ e-learning time accounted for over 50% of their Internet time before the intervention, but the proportion increased to 44.68% (*P* < 0.01) after the course. The weekly log times in the Moodle platform increased significantly after the intervention (*P* < 0.01). Before the intervention, only 18.26% of students logged in to Moodle no fewer than five times per week. The proportion ultimately rose to 39.36%. When they encountered learning difficulties, more participants said that they would like to seek solutions from Moodle (*P* < 0.05), including creating a new discussion post or contacting the tutor directly. The majority of students (90.43%) could complete the homework on time. The proportion increased to 96.81% after the course (see Table 4).

**Table 4:** Students’ e-learning behaviour (*n*, %)

Item	Classification	Before (*N* = 115)	After (*N* = 94)	*x* ^2^	*P*
Time on e-learning/the Internet	<10%	16 (13.91)	8 (8.51)	9.49	0.0021§
	10%–30%	43 (37.39)	29 (30.85)		
	30%–50%	35 (30.43)	15 (15.96)		
	>50%	21 (18.26)	42 (44.68)		
Number of weekly logins in Moodle	≤1	17 (14.78)	7 (7.45)	10.10	0.0015¶
	2–4	77 (66.96)	50 (53.19)		
	5–7	16 (13.91)	37 (39.36)		
	>7	5 (4.35)	0 (0.00)		
How to deal with difficulties	Without the aid of Moodle	80 (69.57)	51 (54.26)	5.18	0.0228
	With the aid of Moodle	35 (30.43)	43 (45.74)		
Whether the tasks on Moodle were completed on time	Basically not	2 (1.74)	1 (1.06)	3.29	0.0695
	Occasionally	9 (7.83)	2 (2.13)		
	Almost certainly	104 (90.43)	91 (96.81)		
Homework assignments	Not serious	1 (0.87)	0 (0.00)	2.79	0.0946
	Serious	56 (48.70)	36 (38.30)		
	Very serious	58 (50.43)	58 (61.70)		

§We applied multiple comparisons on the basis of an adjusted α = 0·0083: *p* = 0·5470 for “<10%” versus “10%-30%”, *p* = 0.7733 for “<10%” versus “30%-50%”, and *p* = 0052 for “<10%” versus “>50%”; *p* = 0·2469 for “10%-30%” versus “30%-50%”, *p* = 0.0023 for “10%-30%” versus “>50%”, and *p* = 0·0001 for “30%-50%” versus “>50%”;

¶We applied multiple comparisons on the basis of an adjusted α = 0·0083: p = 0·3459 for “≤1” versus “2–4”, *p* = 0.0009 for “≤1” versus “5–7”, and *p* = 0·1731 for “≤1” versus “>7”; *p* = 0·0002 for “2–4” versus “5–7”, *p* = 0.0762 for “2–4” versus “>7”, and *p* = 0·0021 for “5–7” versus “>7”

### Multivariate analysis of e-learning KAP

Based on the survey, the entropy method was used to calculate the summed scores of each part. Before the multivariate analysis, we divided the 115 students who participated in the first survey into two groups. Of those students, 22 were lost at follow-up, whereas the others finished the second survey. The summed scores of each part before the intervention were compared between the two groups, and no significant differences were found.

The results of the multivariate analysis are shown in Tables 5, 6 and 7.

**Table 5:** Multivariate analysis of e-learning knowledge

Effect	Classification	Estimate	*t(P)*
Intercept		59.18	15.44 (<.0001)
Educational reform	Before	−6.45	−4.99 (<.0001)
	After	0.00	
Self-perceived Character	Introversion type	−2.05	−0.73 (0.466)
	Extroversion type	−6.91	−2.6 (0.0101)
	Middle type	0.00	
Registered permanent residence	Urban	5.35	2.63 (0.0094)
	Rural	0.00	
Hours spent online per day	Less than 1 h	−11.34	−2.28 (0.0236)
	1 to 3 h	−8.77	−2.35 (0.0200)
	3 to 5 h	−8.93	−2.26 (0.0251)
	More than 5 h	0.00

**Table 6:** Multivariate analysis of e-learning attitudes

Effect	Classification	Estimate	*t(P)*
Intercept		51.97	11.09 (<.0001)
Educational reform	Before	−5.10	−3.4 (0.0010)
	After	0.00	
Community number	0	−5.97	−1.63 (0.1046)
	1–2	−1.00	−0.31 (0.7554)
	≥3	0.00	
Internet age	≤6 years	−7.00	−2.76 (0.0065)
	7–9 years	1.81	0.71 (0.4792)
	≥10 years	0.00	
Hours spent online per day	Less than 1 h	−2.39	−0.45 (0.6506)
	1 to 3 h	5.83	1.49 (0.1386)
	3 to 5 h	3.98	0.96 (0.3384)
	More than 5 h	0.00

**Table 7:** Multivariate analysis of e-learning practices

Effect	Classification	Estimate	*t(P)*
Intercept		51.97	11.09 (<.0001)
Educational reform	Before	−5.10	−3.4 (0.0010)
	After	0.00	
Community number	0	−5.97	−1.63 (0.1046)
	1–2	−1.00	−0.31 (0.7554)
	≥3	0.00	

Educational reform, self-perceived character, registered permanent residence and hours spent online per day were significant impact factors of e-learning knowledge, increasing the average score 6.45 points (*P* < 0.0001) during the intervention. Regarding e-learning attitudes, educational reform, community number, Internet age and hours spent online per day were significant impact factors, increasing the average score 5.10 points (*P* = 0.0010) during the intervention. Regarding e-learning behaviour, educational reform and parents’ literacy were significant impact factors, increasing the average score 10.05 points (*P* < 0.0001) during the intervention.

## Discussion

This study found that the participants did not know much about the concept of Web2.0. The functions of Internet TV that were listed in the survey were equivalent to the user’s privileges in the Web2.0 environment. Nearly half of the students were aware of all the functions. Ten applications also reflected the concept of Web2.0. Most of the students understood more than five. Although the respondents knew little about Web2.0, they were familiar with its functions and applications. Therefore, the participants had good background knowledge of online learning. After the first class, more than 90% of the students acquired seven basic operations, which indicated a strong learning ability.

The educational intervention that combined Moodle with a traditional classroom achieved good results in this study, as reflected in the following aspects. For example, more participants acknowledged that Moodle helped a great deal in learning and were more willing to use Moodle. Additionally, the ratio of e-learning time to Internet time increased significantly, and more participants were willing to seek solutions from Moodle when they encountered learning difficulties. These results are in line with the findings of other studies [26, 27]. Novo-Corti and colleagues [26] suggested that blended learning clearly promotes participation among students, increases their motivation and improves their competence. Martin-Blas and Serrano-Fernandez [27] used Moodle as a teaching tool in physics and found an increasing in the total number of daily visits during the course.

Du and Li [28] divided the impact factors of online learning behaviour into learners’ individual characteristics and environmental factors. This study examined pedagogical reform, gender, community number, self-perceived character, registered permanent residence, parents’ literacy, Internet age, and hours spent online per day. Multivariate analyses indicated that self-perceived character, registered permanent residence, and hours spent online per day had a significant impact on e-learning background knowledge and e-learning ability in addition to pedagogical reform. Introversion and middle type respondents’ average scores were higher than extroversion type respondents’ scores. Although extroverts perform well in face-to-face communication, this advantage works against them when the communication changes from on-site to online. In contrast, introversion and middle type respondents can utilize the advantage of their introspective and independent character in non-face-to-face communication and avoid the disadvantages of poor performance in on-site communication [29].

The multivariate analyses of e-learning attitudes showed that participants whose Internet age was no more than 6 years scored 7.00 points lower than those whose Internet age was more than 10 years. Calculated from time, the former connected to the Internet at the university, whereas the latter began to be influenced by the Internet when they were in junior high school. Therefore, they witnessed the surprisingly rapid development of the Internet during the last ten years, knew the tremendous potential of the Internet and deeply approved of the importance of the network for learning.

## Conclusions

This educational intervention that combined Moodle with a traditional class achieved good results in terms of students’ e-learning KAP. In addition, self-perceived character, Internet age, and hours spent online per day were significant impact factors of e-learning KAP. This type of blended course might be implemented in many other curriculums.

## Limitations and strengths

The major limitation of our study is the absence of a control group. The educational intervention was created based on the original teaching plan of the university. In addition, only one statistics class was developed. We might divide the participants into two classes in the future to acquire a comparative teaching outcome of blended learning.

An important strength of the study is that we fitted a mixed linear model to analyse the impact of factors of KAP. Although nearly 20 participants were lost at follow-up at the end of the curriculum, a mixed linear model can utilize the data without imputation.

A second strength is that the summed scores for every part (knowledge, attitude and practice) were calculated using the entropy method. The entropy value is a measure of the degree of disorder of system information and can measure the amount of useful information in the data [30]. When the data on one object show large differences, according to information theory, the entropy would be low. This shows that the object could contribute a great deal of useful information. Therefore, its weight should be set high. Otherwise, the weight should be set low correspondingly [31]. The entropy weighting method is objective.

## Abbreviations

KAP: Knowledge, attitude and practice
Moodle: Modular object-oriented dynamic learning environment
N: Number
